# Giant cystic lymphangioma of the small bowel mesentery: case report

**DOI:** 10.4314/pamj.v9i1.71228

**Published:** 2011-08-27

**Authors:** Khalid Khattala, Mohamed Rami, Aziz Elmadi, Abdelhalim Mahmoudi, Youssef Bouabdallah

**Affiliations:** 1 Department of Pediatric Surgery, University of Hospital, Fez, Morocco

**Keywords:** Giant cystic lymphangioma, abdominopelvic mass, imaging, surgery, Morocco

## Abstract

Cystic lymphangioma is an uncommon mesenteric tumor which is usually reported in children. We describe a case of a 12-years old girl who was admitted in our hospital with abdominal distension and pain. Physical examination showed an abdomino-pelvic mass. Preoperative studies including abdominal ultrasonography and computed tomography failed to determine the cause of the pain. Laparotomy found a giant cystic tumor of the small bowel mesentery. Histological studies confirm the diagnosis of cystic lymphangioma. Based on this case, a review of the literature is suggested.

## Introduction

Cystic lymphangioma is an uncommon mesenteric, benign slow growing tumor derived from the lymphatic vessels. It is rarely found as intraabdominal mass when occurring in the abdomen [1,2]. Abdominal ultrasonography, CT-scan and celioscopy might be useful for establishing the diagnosis [3,4]. The treatment is mainly surgical and consists of enucleation when feasible. Segmental intestinal resection is achieved when the cyst adheres intimately to the bowel [2,5]. We describe a giant cystic lymphangioma in a 12-years girl who was treated surgically with very good outcome.

## Case report

**Figure 1:**
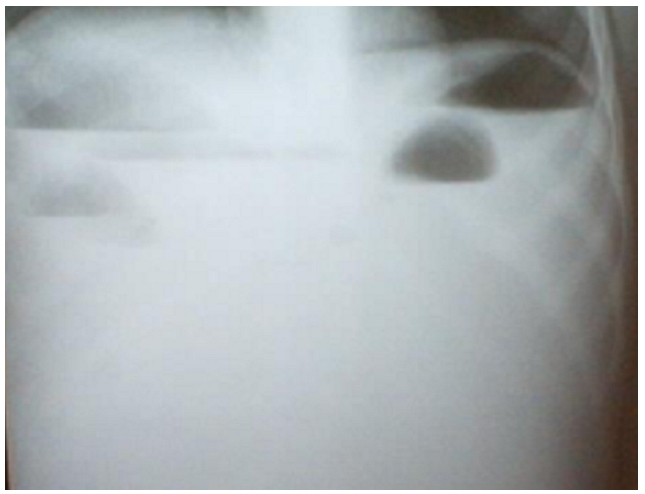
X-ray showing a hydroaeric level driven back to the periphery

**Figure 2:**
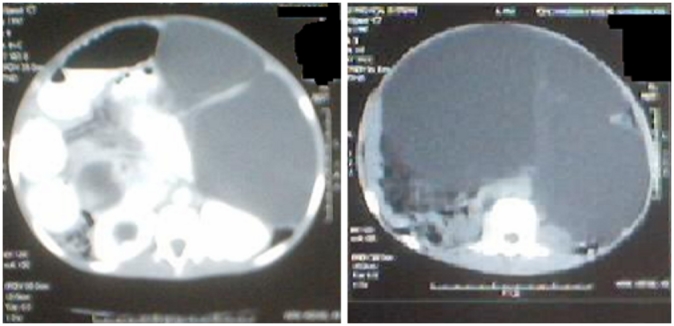
The CT- scan demonstrating a cystic mass

**Figure 3:**
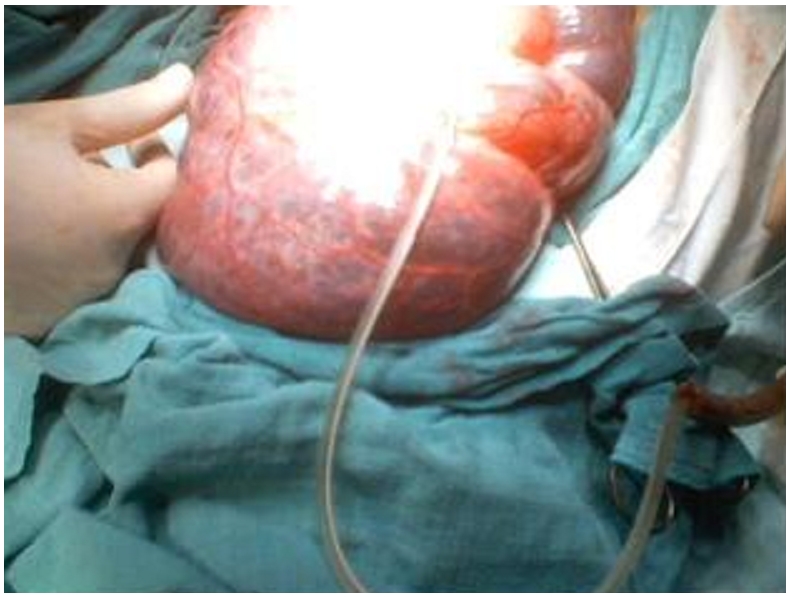
A giant mesenteric tumor was discovered using laparotomy, it contained 5 liters of clear fluid which was emptied

**Figure 4:**
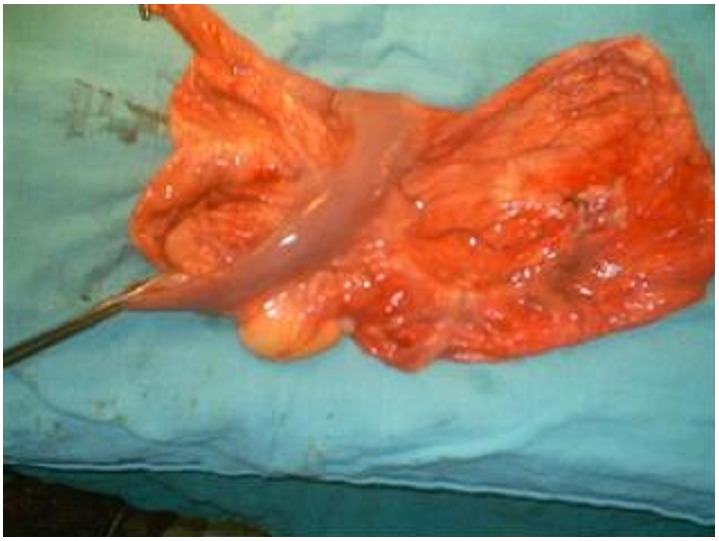
The resected surgical piece with a segment of intestine

A 12 year old female consulted for a clinical profile characterized by nausea, vomiting and abdominal ballottement during eight previous days. This patient reported a history of chronic constipation for which intermittent medical treatment administered for 2 years had failed. Clinical examination found a cachectic child with an extremely distended abdomen, and a palpable abdominopelvic mass. Biological assessments including the blood cell count, alpha-fetoprotein and the beta human chorionic gonadotrophin (B-HCG) were all normal. A full abdominal X-ray showed a bowel loop displaced by a mass in the soft tissues (Figure 1). Ultrasonography revealed an abdominal multiloculated septated cystic mass measuring 20 cm in diameter. CT-scan revealed a septated cystic abdominopelvic mass (Figure 2). The laparotomy was performed, and a huge mesenteric tumor containing 5 litters of clear fluid was discovered (Figure 3), followed by the resection of the involved bowel and the mesenteric tumor ( Figure 4). A primary anastomosis was performed, and the diagnosis of cystic lymphangioma was revealed by histological and immune-histo-chemical studies of the surgical piece. The surgical follow-up was uneventful and the clinical evolution was very favorable 6 years post-surgery.

## Discussion

Cystic cavernous lymphangiomas are uncommon tumors that most often occur in the head, neck, axilla, or groin of young children. Occasionally, the tumor is also discovered in adult in various other anatomic sites [3]. Cystic lymphangiomas is a benign slow growing tumor deriving from lymphatic vessels; it is rarely found as intra-abdominal mass when occurring in the abdomen [1, 2]. Cystic lymphangiomas might arise with acute abdominal pain associated to bowel obstruction, signs of peritonitis [1, 6], chronic abdominal swelling that is detected by palpation of a cystic mass or abdominal swollenness with lower extremities lymphoedema [4, 5]. It is more frequently found in childhood and is mostly diagnosed in the first five years of life [2, 6].

Radiology is the revealing diagnostic tool. Abdominal ultrasonography is the procedure of choice for establishing the diagnosis, even during the antenatal stage [3, 4]. Acute lymphangiomas associating intracystic hemorrhage are more difficult to diagnose; CT-scan and celioscopy might be useful approaches in this context [4]. Sequential ultrasonography and CT-scan examinations show progressive enlargement of the cystic masses, increase of fluid echogenicity and wall thickening associating multiplication of septa [7].

The final diagnosis is always based on histological findings, since this examination shows extensive myofibroblastic areas and determines the lymphatic character of the lesion [5, 6]. The tumor is composed of cystically dilated lymphatic spaces, which are partially invested by a layer of smooth muscle and are associated to occasional lymphoid aggregates. The lymphatic spaces contain either clear fluid or large numbers of foamy macrophages. The lymphatic endothelial cells lining the cystic spaces are generally attenuated without any cytological atypism [6, 7]. The immunohistochemistry showed that the endothelial cells lining the dilated lymphatic spaces are positive for CD31, D2-40, CD34, and negative for keratin [6, 8]. Local recurrence of the tumor is possible [1].

The treatment is mainly surgical and consists of enucleation when feasible; the segmental intestinal resection is achieved when the cyst adheres intimately to the bowel [2, 5]. Few reported cases of diffuse malformation required an extensive bowel resection, which may cause short bowel syndrome [2,7]. The resection could be performed with laparoscopic technique without large incisions [1,6]. Tumors are cystic masses associated to areas of fat necrosis and hemorrhage. Often, cysts contain thick, gelatinous or milky fluid [6]. Sclerosis techniques constitute an interesting alternative and complementary treatment approach, intracystic sclerotherapy using doxycycline is possible for unresecable lymphangiomas [4,8]. The local recurrence of the tumor is possible [1].

## Conclusion

The cystic mesenteric lymphangioma is rare benign tumor. The clinical symptomatology is polymorphic and not specific. The diagnosis is suggested by the imaging modalities, but still requiring a histopathologic confirmation. The treatment of choice is surgical and consists of a full resection of the lesion. Intracystic sclerotherapy could be used for symptomatic tumors associating diffuse mesenteric lesion which are not resecable without extensive intestinal sacrifice.
